# Public Views of Dairy Calf Welfare and Dairy Consumption Habits of American Youth and Adults

**DOI:** 10.3389/fvets.2021.693173

**Published:** 2021-08-11

**Authors:** Rielle K. Perttu, Beth A. Ventura, Aaron K. Rendahl, Marcia I. Endres

**Affiliations:** ^1^Department of Animal Science, College of Food, Agricultural and Natural Resource Sciences, University of Minnesota, St. Paul, MN, United States; ^2^Department of Veterinary and Biomedical Sciences, College of Veterinary Medicine, University of Minnesota, St. Paul, MN, United States

**Keywords:** calf welfare, public views, welfare concepts, consumption habits, parents and children

## Abstract

The primary objective of this study was to explore views of dairy calf welfare and dairy product consumption habits among youth and adults. The secondary objective was to explore views of dairy calf welfare and dairy product consumption habits among a subset of parent-child pairs. Participants 5–17 years of age (*n* = 463) and 18 years old or greater (*n* = 1,310) completed an in-person survey at the Minnesota State Fair (St. Paul, MN, USA) in summer 2018. A subset of these data was comprised of parent-child pairs (*n* = 188). The survey was administered *via* Qualtrics using iPads and included multiple-choice questions about demographics and calf welfare, an open-ended question on “what dairy calves need to have a good life,” and multiple-choice questions about participants' consumption of dairy products and nondairy alternative products. Content analysis was used for responses to the open-ended question, and concepts to describe dairy calf welfare views were identified. Fisher's exact test and Cohen's Kappa were used to investigate the relationships between parent-child pair responses about dairy calf welfare. In addition to these methods, prevalence-adjusted and bias-adjusted kappa (PABAK) were used to investigate the relationships between parent-child pair responses about consumption habits. The median age of all youth participants was 11 years and 61% were female, 82% were urban residents, and 63% did not have prior experience handling agricultural animals but 83% had visited a farm in the past. Most youth participants (94.4%) indicated that they consumed dairy products, while 47.1% consumed nondairy alternatives products. Median age range of all adult participants was 45–54 years, 65% were female, 82% urban residents, and 81% did not have prior experience handling agricultural animals but 63% had visited a farm in the past. Most adult participants (94%) indicated that they consumed dairy products and 47% indicated that they consumed nondairy alternative products. In response to “what dairy calves need to have a good life,” youth and adults most commonly focused on issues related to biological functioning (82 and 70% of youth and adults mentioning this concept, respectively), followed by natural living (44 and 50%, respectively), humane care (30 and 20%, respectively), and affective states (5% of both youth and adults). For the natural living concept of animal welfare, parent and child responses were slightly associated (Kappa = 0.19; *P* = 0.01; overall agreement = 61%). Almost all participants reported consuming dairy products, therefore, the agreement is high between parents and children because in most households (90%), both parents and children consume dairy products. However, child consumption was observed to be lower (75%, 9/12) when parents do not consume dairy than when parents do consume dairy (95%, 167/176), leading to a Kappa of 0.20 (*P* = 0.006, PABAK = 0.81) and a slight association between parents and children. The results suggest that biological functioning is highly valued by the public and views of parents and their children related to natural living in dairy calves are slightly associated.

## Introduction

In a demand-driven economy, consumers play a large role in food production practices (1, 2). We have seen in recent years that citizens have taken their voice to the voting polls, for example to ban certain animal production practices (3). In previous elections, citizens have voted to ban certain livestock housing practices, such as gestation crates and battery cages (4). Today's consumers expect animal products to be produced safely, efficiently, and with attention to the welfare of animals (5, 6). It is therefore of increasing importance to the dairy sector to better comprehend how consumers and other members of the public perceive the welfare of its animals, including the dairy calf (7–9).

Youth views of dairy calf welfare are also of interest as they might influence dietary choices, therefore potentially affecting industry practices (10). Youth stakeholders are often overlooked in their power as industry influencers, even though they are future policy makers and consumers (10). However, to our knowledge, little information is available on youth views toward welfare of the dairy calf. The Social Learning Theory suggests that children develop food preferences and eating habits from observational learning and modeling (11). Other research suggests that parental attitudes toward food products indirectly influence children's food preferences and habits due to exposure to household served foods (12). Other work (13) reported that children's understandings of companion animals is based on age, gender, and parental influence and that “parental attitudes to meat production and consumption influence conversations about meat origins with children” (14).

Some research has focused on impact of animal welfare education on the development of adolescent attitudes toward farm animals (15, 16). However, little is known about how the parent–child relationship may affect the development of one's views toward the welfare of production animals, including the dairy calf. Investigation of parent–child relationships may shed light on the formation of personal views toward agriculture, animal welfare and future consumption of dairy and nondairy products. Previous research suggests that parental influence only slightly moderated children's choices, while media was more likely to influence food choices in children between 3 and 8 years of age (17). However, other work suggests that parental influence on childhood food choices is significant (18). For example, one study (19) found that positive parental influence related to intake of dairy in children may lead children to choose, in addition to dairy, other calcium-fortified foods; however, further research is needed to understand how the parent-child relationship affects a child's consumption choices.

In recent decades, consumption of nondairy plant-based alternative products has increased while fluid milk consumption has decreased (20). If the dairy industry is to increase or maintain current milk sales, a better understanding of why consumers choose dairy or nondairy alternative products is needed. Consumer transitions to nondairy plant-based alternatives appear to be driven by a combination of increased interest in health trends, manufacturer health claims, allergen concerns, and beliefs about environmental and animal welfare impacts (20). Therefore, the objectives of this study were to explore (1) views of dairy calf welfare and dairy product consumption habits of youth and adults; and (2) to explore views of dairy calf welfare and dairy product consumption habits among a subset of parent–child pairs.

## Materials and Methods

We used a mixed-methods survey to investigate dairy calf welfare views and dairy and nondairy alternative product consumption habits among fairgoers attending the 2018 Minnesota State Fair in St. Paul, Minnesota. The University of Minnesota's Institutional Review Board approved the study (including the survey instrument) under protocol #00003443. The Minnesota State Fair has an annual attendance of ~2 million people, and it includes attractions beyond animal barns and agricultural exhibits. Known as the “great Minnesota get-together,” the fair offers many kinds of activities for children and adults, including amusement rides, daily concerts, restaurants, and hundreds of food stands and merchants within its 130 ha. Participants were recruited at the University of Minnesota “Driven to Discover” research building at the fair over five 7-h shifts between August 25 and September 2, 2018. As prospective participants neared our study area within the building, our research team approached them and briefly described the study and its purpose and inquired if they were willing to participate. Parents had to consent to the survey for their child to participate and youth also assented to complete the survey. Youth had to be at least 5 years of age and able to read and write in English to be included in the study. This age group was chosen because children learn to read around 5 years old, on average (21). The survey was anonymous, administered *via* iPads (Apple, Cupertino, CA), and data were collected and managed using Qualtrics survey software (Qualtrics, Provo, UT). Participants were sequentially assigned anonymous IDs upon starting the survey (e.g., PY1 = youth participant #1 and PA1 = adult participant #1, etc.). If participants were a parent–child pair, they were sequentially assigned paired anonymous IDs (PAIR1 = parent-child pair #1). Participants received a small drawstring backpack or a cow-shaped stress-ball upon completion of the survey as incentive to participate.

### Survey Description

Our research team developed a 10-min survey to document adult and youth views of dairy calf welfare and consumption habits of dairy and nondairy alternative products. Questions were developed by RP, BV, and ME and adapted from survey language from Ventura et al. (22). Youth received a modified version of the adult survey containing simplified language (e.g., “Have you ever been on a livestock farm?” became “Have you visited a farm with animals?”). A draft of the survey instrument was piloted among animal science faculty and undergraduate students at the University of Minnesota, St. Paul (due to convenience and availability) and questions were refined as needed based on feedback (i.e., to clarify language or intent behind a question). Adults could observe their children during the survey but were asked not to help them with the survey instrument. Researchers were on hand if youth needed clarification or help completing the survey (e.g., in using the iPad or navigating the Qualtrics platform). The surveys consisted of 12 multiple choice questions and 1 mandatory open-ended question to explain their views of dairy calf welfare. No participants abandoned the survey once it was launched.

The survey instrument included demographic questions, described in detail in our previous work (23), on age, gender, area of residence, prior experience handling agricultural animals, prior experience visiting a farm, having a loved one who works in the dairy industry, and pet ownership. Youth participants had the option to select “*I don't want to say*” for questions asking for their gender, prior experience handling agricultural animals, prior experience visiting a farm, having a loved one who works in the dairy industry, and pet ownership. Adult participants had the option to opt out of answering questions about their age and gender. Finally, youth participants were also asked if they enjoyed consuming dairy and nondairy plant-based alternative food products (“milks”) and adult participants were asked if they consumed dairy and nondairy plant-based “milks.”

Participants were then asked the open-ended question, “*What does a dairy calf need to have a good life?*” accompanied with a picture of a dairy calf on a white background and given a mandatory space (with no character limit) to respond. After completion of the open-ended question, participants were prompted to “*Think about what a dairy calf needs to have a good life. How important do you think these things are?*” and then received three items: (1) *the right amount of food, water, shelter, and doctor care*; (2) *ability to play with other calves;* and (3) *treated calmly and respectfully by their owner*. For these questions, participants were asked to rate their response on a scale ranging from “not important” [1] to “very important” [5].

### Survey Analysis

#### Quantitative Analysis

The SURVEYFREQ procedure (SAS 9.4, Cary, Indiana) was used to estimate the totals and proportions of the categorical variables—gender, age for youth or age range for adults, area of residence, previous experience working with agricultural animals, previous experience visiting a farm, if the participant had a loved one in the dairy industry, previous pet ownership, and consumption habits for dairy and nondairy plant-based alternative products. This procedure was also used to estimate the totals and proportions of categorical responses to the “*Think about what a dairy calf needs to have a good life. How important do you think these things are*?” questions.

Fisher's exact test and Cohen's Kappa were used to investigate the relationship between parent and child responses, for both consumption habits and the concepts of what a calf needs to have a “good” life (see section Qualitative Analysis, below). The *p*-values from Fisher's exact test were used for the hypothesis test and Cohen's Kappa was used to understand the level of agreement relative to chance. The agreement reported is characterized by a commonly used scale such as the Landis and Koch (24). The interpretation used for Cohen's Kappa was as follows: poor agreement below 0.20, fair from 0.21 to 0.40, moderate from 0.41 to 0.60, substantial from 0.61 to 0.80, and almost perfect agreement from 0.81 to 1.00 (24). The prevalence-adjusted bias-adjusted kappa (PABAK) was examined for consumption habits because it gives an indication of the likely effects of prevalence and bias index (25). *P* < 0.05 were considered significant. These calculations were performed in R version 4.0.2 (26).

#### Qualitative Analysis

Content analysis was applied to participant responses about what a calf needs to have a “good” life. This process began with thoroughly reading, re-reading, and coding all text from the responses for emerging patterns (27). In this process, we identified and labeled phrases or statements within the free-text data contributed by participants with code labels to describe like phrases or statements with similar meaning. RP coded all responses independently and then discussed the preliminary codes with ME and BV. A finalized codebook was created once all authors agreed on preliminary codes. Data were coded deductively into concepts of Fraser et al.'s (28) animal welfare framework in terms of (1) biological functioning (emphasis on physical condition of the animal and overall health, including references to food, water, shelter, hygiene, and safety), (2) natural living (emphasis on the calf's ability to live naturally, meaning calves are in perceived naturalistic environments and have the opportunity to exercise natural behaviors), and (3) affective state (emphasis on the animal's mental or emotional state, focusing both on calves being able to experience positive states as well as avoid negative states). Ultimately, the final coding scheme was expanded beyond this framework based on participants' responses to incorporate an additional concept of humane care (elements related to care and attention provided by humans) following previous work by Ventura et al. (22). After all coding was complete, the percentage of participants referencing each concept was calculated. Example responses that demonstrate concepts are quoted below, followed by participant number in brackets (e.g., _[PA12]_ to designate Adult Participant #12 or _[Parent 12]_ to designate parent of Adult-Child Pair #12).

## Results

### Description of Participants: Youth

A total of 463 youth participants completed the survey and were included in the final analysis, though a smaller proportion chose to report information on their gender (*n* = 334) and area of residence (*n* = 333). The median age of participants was 11 years, and the majority of participants were female (61%), had lived most of their lives in urban or suburban settings (82%) and had owned a pet in the past or currently owned a pet (90%). Over three-fourths had visited a farm (76%) but had not regularly worked with or handled farm animals (63%), nor did they have loved ones who worked in the dairy industry (76%). The majority of participants (94%) indicated that they consumed dairy products, while 47% consumed nondairy alternatives such as almond, soy, or other plant-based beverages.

### Description of Participants: Adults

A total of 1,310 adult participants completed the survey and were included in the final analysis. The majority of participants identified as female (65%), 35% as male, and 0.3% as gender non-conforming or transgender. The median age range was 45–54 years and 82% had lived most of their lives in an urban or suburban setting. Most (81%) participants had not worked with or handled farm animals, but 63% had visited a farm with animals. Additionally, 79% of the participants did not have a loved one who worked in the dairy industry and 94% had owned a pet in the past or currently owned a pet. Most participants (94%) indicated that they consumed dairy products, while 47% consumed nondairy alternatives such as almond, soy, or other plant-based beverages.

### Description of Participants: Parent–Child Pairs

Within this participant population, a total of 188 parent-child pairs completed the survey and were included in the final analysis; 147 of these children chose to report information on their gender and area of residence. The median age of children in these pairs was 10 years and the majority were female (60%), had lived most of their lives in urban or suburban settings (84%) and had owned a pet in the past or currently owned a pet (90%). Most had previously visited a farm with animals (82%) but had not regularly worked with or handled farm animals (60%), nor did they have loved ones who worked in the dairy industry (86%).

Most parents in the pairs were female (70%), the median age range was 34–44 years, and 79% had lived most of their lives in an urban or suburban setting. The majority (94%) had owned a pet in the past or currently owned a pet. Most had previously visited a farm with animals (71%) but had not regularly worked with or handled farm animals (81%), nor did they have loved ones who worked in the dairy industry (79%).

### Relationship Between Parent–Child Consumption Habits of Dairy and Nondairy Products

In response to consuming dairy products, 94% (176/188) of parents and 94% (176/188) of children indicated that they consumed dairy products (e.g., milk, cheese, yogurt, butter, or ice-cream). Of the children whose parents indicated that they consumed dairy products, 95% (167/176) also consumed dairy products; of the children whose parents did not consume dairy products, 75% (9/12) of their children did anyway. Therefore, this gives an overall agreement of 90% between the parent-child pairs and a Kappa of 0.20, prevalence-adjusted bias-adjusted kappa [PABAK] 0.81 ([95% CI: 0.71–0.88], *P* = 0.006), suggesting that dairy consumption habits were slightly associated after adjusting for influences of bias and prevalence.

In response to consuming nondairy alternative products, 51% (95/188) of the parents indicated that they consumed nondairy alternatives such as almond, soy, or other plant-based beverages and 47% (88/188) of children also indicated that they consumed nondairy alternative products. Of the children whose parents indicated that they consumed nondairy alternative products, 54% (51/95) also consumed nondairy alternative products; of the children whose parents did not consume nondairy alternative products, 40% (37/93) did anyway. Therefore, this gives an overall agreement of 57% between the parent–child pairs and a Kappa of 0.14, prevalence-adjusted bias-adjusted kappa [PABAK] 0.14 ([95% CI: −0.01 to 0.28], *P* = 0.056) suggesting that nondairy alternative consumption habits were slightly associated after adjusting for influences of bias and prevalence.

### Views of Dairy Calf Welfare: Youth

**Figure 1:**
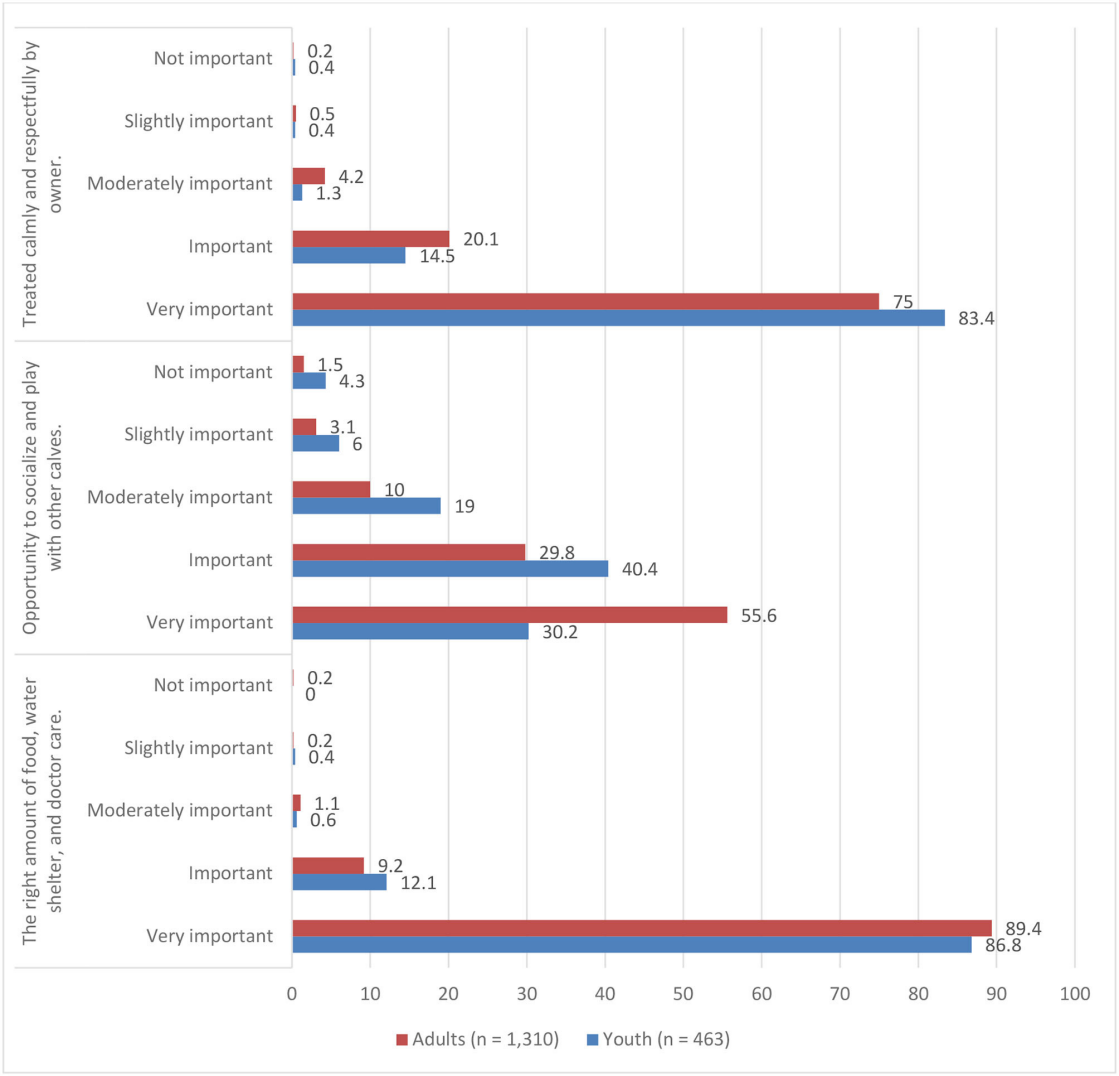
Youth and adult responses to the prompt, “*Think about what a dairy calf needs to have a good life. How important do you think these things are?*” (% of respondents).

Of the youth participants (*n* = 463), nearly all rated the right amount of food, water, shelter, and doctor care, along with being treated calmly and respectfully, as important or very important to calf welfare (90 and 98%, respectively). Nearly three-fourths of youth (71%) rated the ability to play with other calves as important or very important to calf welfare (Figure 1).

In response to what dairy calves need to have a “good” life, youth most commonly mentioned concepts related to biological functioning (82% of responses), followed by natural living (44%), humane care (30%), and affective state (5%). Examples of participant responses were as follows:

(1) Biological functioning: “*water, a healthy diet, shelter to protect them from the weather and predators*” _[PY25]_ and “*good environment with clean bedding*” _[*PY*465]_.
(2) Natural living: “*exercise*” _[PY51]_, “*plants*” _[PY433]_, and “*a calf needs to have a pasture of grass for it and its friends/brothers/sisters to get fresh air*” _[*PY*371]_.
(3) Humane care: “*good caregiver*” _[PY1]_, “*love*” _[PY11]_, and “*it needs a good home and owners…once it grows up it needs to be carefully treated so it doesn't get hurt while being milked, it always needs to be cared for*” _[*PY*190]_.
(4) Affective state: “*leisure time*” _[PY231]_, “*psychological care*” _[PY310]_, and “*[s]he needs to have fun like any other living species*” _[*PY*206]_.

Most youth participants included elements related to more than 1 concept in their responses, with a median value of 1.62 (range: 0–4). For example, the comment “*love, food, water, happiness, room to roam, and comfortable living environment*” _[PY359]_ references biological functioning (*food, water, and comfortable living environment*), natural living (*room to roam*), affective state (*happiness*), and humane care (*love*).

### Views of Dairy Calf Welfare: Adults

Of the adult participants (*n* = 1,310), almost all rated the right amount of food, water, shelter, and doctor care, along with being treated calmly and respectfully, as important or very important to calf welfare (99 and 95%, respectively). Most (85%) rated the ability to play with other calves as important or very important to calf welfare (Figure 1).

Adult participants most commonly mentioned concepts related to biological functioning (70% of responses) in response to what dairy calves need to have a “good” life, followed by natural living (50%), humane care (20%), and affective state (5%). Examples of adult responses included:

(1) Biological functioning: “*food, shelter, warmth, and water*” _[PA155]_, “*dry barn and clean bedding*” _[PA1321]_ and “*a safe farm that they are fed proper supplements at*” _[*PA*474]_.
(2) Natural living: “*fresh air, fresh grass, space to move, sunlight*” _[PA292]_, “*open pasture*” _[PA420]_, “*friends and family*” _[PA980]_, “*social interactions and exercise*” _[PA1179]_ and “*playtime*” _[*PA*352]_.
Humane care: “*love*” _[PA213]_, “*caring farm hands*” _[PA327]_ and “*compassionate farmers*” _[*PA*658]_.
(3) Affective state: “*comfort*” _[PA293]_, “*calm*” _[PA745]_, “*content*” _[PA665]_, “*stress free*” _[PA45]_ and “*so they are not depressed*” _[*PA*818]_.

Most adults referenced more than one concept in their responses, with a median value of 1.45 (range: 0–4). For example, the comment “*clean environment with access to pasture, good food, and good care*” _[PA902]_ referenced biological functioning (*clean environment, good food*), natural living (*access to pasture*) and humane care (*good care*).

### Relationship Between Parent–Child Views of Dairy Calf Welfare

In response to what dairy calves need to have a “good” life, 80% (151/188) of parents and 91% (171/188) of children referenced elements of biological functioning. Of the children whose parents mentioned the concept of biological functioning, 92% (139/151) also mentioned it; of the children whose parents did not, 87% (32/37) did anyway. Therefore, this gives an overall agreement of 77% (144/188) between parent–child pairs; however, this was not significant, with a Kappa of 0.07 indicating slight agreement (*P* = 0.34).

Overall, 62% (116/188) of parents mentioned the concept of natural living and 55% (104/188) of children also mentioned this concept. Of the children whose parents referenced natural living, 63% (73/116) also mentioned it; of the children whose parents did not, 43% (31/72) did anyway. Therefore, this gives an overall agreement of 61% (114/188) between parent-child pairs; this was more than would be expected by chance, with a Kappa of 0.19 indicating slight agreement (*P* = 0.01).

Relative to humane care, 26% (49/188) of parents mentioned this concept and 34% (63/188) of children also mentioned this concept. Of the children whose parents discussed humane care, 39% (19/49) mentioned it; of the children whose parents did not, 32% (44/139) did anyway. Therefore, this gives an overall agreement of 61% (114/188) between parent-child pairs; however, this was not significant, with a Kappa of 0.07 indicating slight agreement (*P* = 0.38).

Finally, just 3% (5/188) of parents and 4% (8/188) of children discussed elements related to affective state in their responses overall. Of the children whose parents mentioned the concept, 0% (0/5) mentioned it; of the children whose parents did not, 4% (8/183) did anyway. Therefore, this gives an overall agreement of 93% (175/188) between parents–child pairs; however, this was not significant, with a Kappa of −0.03 indicating no agreement (*P* = 1.0).

These concepts are described as follows, in descending order of frequency:

(1) Biological functioning: “*quality feeds*” _[Parent 133]_ and “*good environment with clean bedding*” _[Child 133]_.
Natural living: “*exercise*” _[Child 72]_ and “*space to roam and grass*” _[Parent 72]_.
(2) Humane care: “*well-trained caretakers*” _[Child 162]_ and “*fair and humane treatment*” _[Parent 162]_.
(3) Affective state: “*fun*” _[Child 116]_, “*none stressed life*” _[Parent 123]_, and “*not being injected with hormones that make them uncomfortable*” _[Parent 145]_.

Most parent–child pairs included elements related to more than 1 concept in their responses, with a median value of 1.71 for parents and 1.84 for children (range: 0–4). For example, the comment “*a good diet, a caregiver, fresh water, space to grow and be happy, friends; cows have best friends and respond better when given a name, let them grow happy and they provide better*” _[Child 33]_ references biological functioning (*a good diet, fresh water, and grow*), natural living (*space, friends*), affective state (*happiness*), and humane care (*caregiver*). The accompanying parent of the pair said, “*room to roam, being with their mother for an appropriate time, good food and good treatment*” _[Parent 33]_ which references biological functioning (*good food*), natural living (*room to roam, being with their mother for an appropriate time*), and humane care (*good treatment)*.

## Discussion

### Views of Dairy Calf Welfare

Most of the literature on public perspectives of farm animal welfare focuses on adults, with relatively few studies focusing on youth (10, 29); however, youth perspectives are also likely to be relevant to the understanding of how societal conversations and demands for animal welfare arise, in part because children are a critical part of family purchasing dynamics (30). Recent work suggests that parents spend more at the supermarket when children accompany them and that children influence up to 20% of all household purchase decisions (31). Therefore, the objective of the current research was to explore views about dairy calf welfare and dairy and nondairy consumption habits among parents and children.

Previous research has identified that members of the public are concerned about the biological functioning of farm animals, most notably highlighting basic necessities like food, water, shelter, and veterinary care as extremely important for animal well-being (32, 33). The current findings confirm that these attributes are highly prioritized by members of the public, as both youth and adults most frequently referenced biological functioning in their responses to what dairy calves need for a good life.

Aspects relevant to affective state were less commonly raised when participants were asked to identify attributes that are necessary for a dairy calf to have a good life. In our study, youth were more likely to reference the concept of affective state compared to adult participants. This could potentially be linked to young children's high-quality relationships with pets (34). However, children's attachment with animals may be age dependent because research suggests that older children may relax their attachment to pets once they undergo puberty. Patterns of pet attachment in younger children coincide with children's emotional concerns for animals (35, 36) which could potentially explain why more youth than adults mentioned concepts related to affective state in their responses. Additionally, no parent-child pairs both referenced affective state in their responses. The lack of references to affective state overall is likely due to our question framing (rather than to participants' lack of beliefs in the importance of this element), which may have primed participants to think about external stimuli rather than the calf's internal state (22). In our previous work (23), we reported that affective state was mentioned less frequently (compared to biological functioning or natural living) in support of pair and group housing of dairy calves. Other research found that lay citizens consider animals' basic needs related to biological functioning such as feeding, health, and appropriate facilities as the most important aspect of animal welfare (37) while other studies demonstrate lay citizens valuing animal welfare characteristics related to affective state and naturalness (38, 39).

When asked to articulate what a dairy calf needs to have a “good” life, about a quarter of adults and a third of youth found calf welfare to relate to actions of their human caretakers. The findings from our research further emphasize that the public places value on farm animals being treated with care, respect, and affection. Other studies have also found that the public views humane care, gentle handling, and farmer-animal interaction as an important and distinct contributor to the quality of life of the animal (32, 40). For example, Cardoso et al. (39) demonstrated that Brazilian citizens expressed that quality of animal treatment was vital in their visions of the ideal dairy farm. Other research has reported citizens valuing humane care for other livestock industries as well, including swine (41), poultry (42), and beef operations (43).

Finally, both youth and adults also commonly referenced aspects related to natural living in their responses to what calves need for a “good” life, and most participants also rated opportunities for the dairy calf to socialize with other calves as important or very important. It is known that members of the public desire aspects related to natural living to be present on the modern dairy farm (3, 37) and that people express concern that farms inhibit animals from expressing natural behaviors (1, 44). Our findings contribute to the growing literature suggesting that the public places priority on natural living in order for farm animals to have a good life (6, 22, 45).

Overall, this study provides insight about the expectations of American youth and adults and their views of dairy calf welfare. Most participants mentioned at least one concept of animal welfare (biological functioning, affective state, and natural living) along with humane care. When we explored animal welfare views among parent–child pairs, we found parent–child pairs had slight agreement on all concepts of animal welfare; however, only the natural living concept of animal welfare was found to be significantly associated between parent and child responses. For no other concepts (biological functioning, affective state, and humane care), were parent and child responses found to be associated.

Previous research suggests that children are more likely to focus on aspects related to biological functioning, such as an animal's dietary needs, due to children's knowledge of pet care (16). To our knowledge, little research is available on relationships between parental-child views of animal welfare. Research suggests that animal welfare beliefs are affected by culture, age, school year group, and having a companion animal at home (46) but how familial connections may impact these beliefs remains underexplored.

### Dairy Consumption Habits of Parents and Children

It is argued that parents influence children's behaviors toward food and facilitate consumption of certain food products (47), as described by Jung et al. (48): “*parents are gatekeepers of familial nutritional intake and represent a potential vehicle through which to increase dairy consumption in children*.” The results of our study contribute to evidence that family relationships are slightly associated with the consumption of dairy and nondairy alternative products.

Previous research suggests that a parent/main caretaker's gender may play a role in attitudes related to consumption of animal food products such as meat, with women more supportive of children choosing a vegetarian lifestyle and more likely to discuss healthy eating habits with their children (14). Other research has suggested that women generally demonstrate greater empathy and concern for farm animal well-being (49) and are more likely to feel a moral obligation to protect animals compared to men (50). Another study suggests that parents indirectly influence children's food preferences and habits due to availability of foods in the household further emphasizing the impact of parental attitudes toward food products (12). Our research contributes to the evidence that family relationships are associated with the consumption of dairy products and tended to be associated with consumption of nondairy alternative products suggesting that exposure to household foods contributes to food preferences in youth.

Other factors such as the child's age and area of residence are also associated with conversations about food origins with children (14). Additionally, urban parents were more likely to reveal their hesitation with meat consumption and more likely to purchase products marketed from small sustainable farms and free-range due to beliefs of high-standards of animal welfare (14). Another study (47) reported that parents use a variety of parenting practices, beyond parental pressure and dietary restriction, to promote consumption of healthy foods, but there are limited data reporting parents' choices to explicitly choose nondairy alternative products, occurrences that stimulate children's decisions regarding dietary choices or the context in which decisions are made for consuming food products in children. Further research is needed to clarify beliefs that would lead parents to purchase nondairy alternative products compared to dairy products and to explore the role that culture has on children's dairy and nondairy alternative product consumption.

Recent research (51) suggests that both dairy and nondairy alternative products have perceived nutritional benefits, such as calcium, protein, and fat content, which encourages parents to incorporate both options into their child's diet; however, barriers include environmental impact, cost, and high sugar content. That work found that parents opt for child-friendly dairy options due to taste, familiarity, variety, and accessibility of products but are concerned with dairy farming practices and antibiotic and hormone use (51). In contrast, it is perceived that nondairy alternative products add variety to the diet but specific barriers include concerns about the use of pesticides on farms (51). In addition to perceptions affecting parent purchasing decisions, income, and geographical location of their usual food store, point-of-purchase, and store layout may also influence choices for dairy or nondairy alternative products (52). Other factors influence consumer attitudes, such as subjective norms, health consciousness, taste, knowledge, environmental concern, animal treatment, and appearance, while purchase intention is affected by price and curiosity (53). Another study (54) found that similar factors were considered when purchasing dairy or nondairy alternative products and that consumption patterns between children and parents are consistent in households; however, the substitution effects of animal welfare views on consumption patterns differ. The current findings confirm that the parent-child relationship was slightly associated with consumption of dairy products and also tended to be slightly associated with the consumption of nondairy alternative products.

### Study Limitations

Our survey sample was drawn from members of the general public at the Minnesota State Fair who chose to visit the University of Minnesota's “Driven to Discover” building. As such, our participants may be especially supportive of research and education activities, which could have influenced particular responses. However, our survey demographics do align with similar proportions of urban residents in the state of Minnesota (55). We acknowledge that our study was a convenience sample and that we did not capture race or ethnicity demographics, which limits generalization to the US population. Repeating this survey at other events, in other geographic locations, would be valuable in expanding our understanding of the American public's views toward dairy calf welfare.

## Conclusion

To our knowledge, this is the first study to explore views of dairy calf welfare among members of the American public with attention to youth, and to explore specifically how views and consumption of dairy and nondairy alternative products may associate between children and parents. Children were slightly more likely to consume dairy products if their parents also consumed these products. We suggest that adopting management strategies that promote behaviors related to the natural living concept of animal welfare is likely to be viewed positively by members of the American public, and hence will benefit the dairy sector's public image.

## Data Availability Statement

The raw data supporting the conclusions of this article will be made available by the authors, without undue reservation.

## Ethics Statement

The studies involving human participants were reviewed and approved by University of Minnesota's Institutional Review Board. Written informed consent to participate in this study was provided by the participants' legal guardian/next of kin.

## Author Contributions

RP, BV, and ME designed and conducted the study. ME was the PI and coordinated the study. RP and AR conducted the quantitative portion of the statistical analysis. RP led the qualitative analysis and wrote the initial draft of the manuscript. All authors edited, reviewed, and approved the final manuscript.

## Author Disclaimer

The views expressed in this article are those of the authors and do not necessarily represent those of the University of Minnesota.

## Conflict of Interest

The authors declare that the research was conducted in the absence of any commercial or financial relationships that could be construed as a potential conflict of interest.

## Publisher's Note

All claims expressed in this article are solely those of the authors and do not necessarily represent those of their affiliated organizations, or those of the publisher, the editors and the reviewers. Any product that may be evaluated in this article, or claim that may be made by its manufacturer, is not guaranteed or endorsed by the publisher.
