# Supplementary material for: Public Views of Dairy Calf Welfare and Dairy Consumption Habits of American Youth and Adults
Source: Front Vet Sci. 2021 Aug 11;8:693173. doi: 10.3389/fvets.2021.693173 (PMC8385635; doi:10.3389/fvets.2021.693173)
Supplement: Supplementary file 1 [file Table_1.docx]

# **Appendix**

## Youth Survey

Participant Number: _______

Q1. How old are you? _______

Q2. What is your gender?

Girl

Boy

I don’t want to say

Q3. Do you live in the country or the city?

City/suburbs

Country side

Q4. Have you ever worked with or handled farm animals?

Yes

No

I don’t want to say

Q5. Have you visited a farm with animals?

Yes ­­­­­­

No

I don’t want to say

Q6. Do any of your loved ones work in the dairy industry or on a dairy farm?

Yes

No

I don’t know

Q7. Have you ever had a pet?

Yes

No

I don’t want to say

Q8. Do you enjoy eating/drinking dairy products like milk, cheese, yogurt, butter, or ice cream?

Yes

No ­­­­­­

Q9. Do you enjoy eating or drinking any of the following products: almond milk, soymilk, other plant-based milks?

Yes

No

I don’t want to say

Q10. In your opinion, what does a dairy calf need to have a good life?

Q11-13. Think about what a dairy calf needs to have a good life. How important do you think these things are?

The right amount food, water, shelter, and doctor care.

Very important

Important

Moderately important

Slightly important

Not important

Ability to play with other calves.

Very important

Important

Moderately important

Slightly important

Not important

Treated calmly and respectfully by owner.

Very important

Important

Moderately important

Slightly important

Not important

## Adult Survey

Participant Number: _______

Q1. Gender:

Female

Male

Non-binary/other

Prefer not to say

Q2. Age:

18-24

25-34

35-44

45-54

55-64

65-74

75 years or older

Prefer not to answer

Q3. Which best describes where you have lived for most of your life?

City/suburbs

Country side

Q4. Have you ever worked on a livestock farm?

Yes

No

Prefer not to answer

Q5. Have you ever been on a livestock farm?

Yes ­­­­­­

No

Prefer not to answer

Q6. Do any of your loved ones work in the dairy industry or on a dairy farm?

Yes

No

I don’t know

Q7. Have you ever had a pet?

Yes

No

I don’t want to say

Q8. Do you consume dairy products?

Yes

No

Q9. Do you consume any of the following products: soymilk, almond milk, flaxseed milk, or other plant-based milk?

Yes

No

Q10. Consider the dairy calf: in your opinion, what do dairy calves need to have a good quality of life?

Q11-13. Consider the following aspects of dairy calf’s life. Please let us know how important they are to you:

Adequate food, water, shelter, and veterinary care.

Very important

Important

Moderately important

Slightly important

Not important

Opportunity to socialize (within a week after birth) and play with other calves.

Very important

Important

Moderately important

Slightly important

Not important

Being treated calmly by caretaker to avoid causing fear and distress.

Very important

Important

Moderately important

Slightly important

Not important
